# Everolimus improves neuropsychiatric symptoms in a patient with tuberous sclerosis carrying a novel *TSC2* mutation

**DOI:** 10.1186/s13041-016-0222-6

**Published:** 2016-05-23

**Authors:** Su-Kyeong Hwang, Jae-Hyung Lee, Jung-eun Yang, Chae-Seok Lim, Jin-A Lee, Yong-Seok Lee, Kyungmin Lee, Bong-Kiun Kaang

**Affiliations:** Department of Pediatrics, Kyungpook National University Hospital, Daegu, 41944 South Korea; Department of Life and Nanopharmaceutical Sciences, Department of Maxillofacial Biomedical Engineering, School of Dentistry, Kyung Hee University, Seoul, 02447 South Korea; Department of Biological Sciences, College of Natural Sciences, Seoul National University, Seoul, 08826 South Korea; Department of Biotechnology and Biological Sciences, Hannam University, Daejeon, 34430 South Korea; Department of Life Science, Chung-Ang University, Seoul, 06974 South Korea; Behavioral Neural Circuitry and Physiology Laboratory, Department of Anatomy, Brain Science & Engineering Institute, Kyungpook National University Graduate School of Medicine, Daegu, 41944 South Korea

**Keywords:** Tuberous sclerosis, Autism, Everolimus, Mutation, High throughput nucleotide sequencing

## Abstract

Tuberous sclerosis complex (TSC) is a neurocutaneous disorder characterized by multiple symptoms including neuropsychological deficits such as seizures, intellectual disability, and autism. TSC is inherited in an autosomal dominant pattern and is caused by mutations in either the *TSC1* or *TSC2* genes, which enhance activation of the mammalian target of rapamycin (mTOR) signaling pathway. Recent studies have suggested that mTOR inhibitors such as rapamycin can reverse TSC-associated deficits in rodent models of TSC. In addition, clinical trials are ongoing to test the efficacy of mTOR inhibitors toward the psychiatric symptoms associated with TSC. Here, we report a case study of a Korean patient with TSC, who exhibited multiple symptoms including frequent seizures, intellectual disability, language delays, and social problems. We performed whole exome sequencing and identified a novel small deletion mutation in *TSC2*. Expressing the novel deletion mutant in HEK293T cells significantly increased mTOR pathway activation. Furthermore, everolimus treatment showed not only reduction in SEGA size, but dramatically improved behavioral deficits including autism related behaviors in the patient. In summary, we identified a novel small deletion mutation in *TSC2* associated with severe TSC in a Korean family that enhances the activation of mTOR signaling in vitro. Everolimus treatment improved behavioral deficits in the patient.

**Electronic supplementary material:**

The online version of this article (doi:10.1186/s13041-016-0222-6) contains supplementary material, which is available to authorized users.

## Introduction

Tuberous sclerosis complex (TSC) is a genetic disorder characterized by the presence of benign hamartomas in any organ system, with highly variable, unpredictable, and potentially devastating neurological outcomes [1, 2]. TSC is the second most common identified neurocutaneous disorder with an estimated incidence of 1:6000, affecting more than 1 million individuals worldwide [3, 4]. The majority of patients have central nervous system involvement that manifests as structural brain abnormalities, epilepsy, and cognitive, behavioral, and psychiatric deficits including autism spectrum disorder (ASD) [5, 6]. All types of seizures are seen, often in combination. Half of individuals have normal intelligence, but almost none are free from neuropsychiatric problems [7]. Importantly, ASD is diagnosed in approximately 40–60 % of patients with TSC, and TSC accounts for 3–4 % of ASD [8–10].

TSC is caused by mutations in either of two tumor suppressor genes, *TSC1* or *TSC2*, encoding hamartin and tuberin, respectively [11]. TSC is inherited in an autosomal dominant pattern and haploinsufficiency of *TSC* causes neurological phenotypes including learning disability and social deficits [10, 11]. *De novo* mutations account for approximately 80 % of TSC cases. *TSC2* mutations are four times as common as *TSC1* mutations among *de novo* cases, whereas the prevalence of *TSC1* and *TSC2* mutations is approximately equal among familial TSC cases [12]. *TSC1* and *TSC2* mutations lead to essentially identical phenotypic manifestations, although there have been some suggestions that the *TSC2* phenotype is typically more severe [13, 14]. The TSC1 and TSC2 proteins act as a heterodimer to suppress mammalian target of rapamycin (mTOR), a serine/threonine protein kinase that regulates cell growth and division [5, 15]. Loss of either *TSC1* or *TSC2*, followed by a “second hit” of the remaining functional allele thereby preventing formation of the heterodimeric complex, causes loss of regulatory control over mTOR and leads to overactive cell growth and proliferation [1, 16]. Thus, at the cellular level, loss of *TSC1* or *TSC2* results in upregulation of the mTOR pathway [10, 17]. The molecular understanding of the TSC pathophysiology has opened up possibilities for molecular targeted treatments of the neuropsychiatric phenotype in TSC using mTOR inhibitors such as rapamycin [16]. Notably, rapamycin treatments have been shown to successfully reverse the deficits in behavior and synaptic plasticity in rodent models of TSC [10, 18–20].

Recently, the mTOR inhibitors everolimus and sirolimus have been shown to exhibit efficacy for the treatment of several manifestations of TSC such as subependymal giant cell astrocytomas (SEGA), seizures, renal angiomyolipomas, lymphangioleiomyomatosis, and facial angiofibroma lesions in patient with TSC [21–24]. Moreover, human and animal studies suggest that mTOR inhibitors improve deficits of sociability, learning and neurodevelopment in TSC mouse models and patients with TSC [18, 25, 26]. On the basis of these findings, although some clinical trials have been completed or initiated to test whether everolimus treatment might improve neurocognition, features of autism, and the neuropsychological deficits in children with TSC (clinicaltrials.gov study ID: NCT01289912, NCT01730209), in the present study we present a case of a family with a novel *TSC2* mutation in which the behavioral phenotypes of a 3-year-old boy with TSC accompanied by severe autism could be dramatically improved by everolimus treatment.

## Results

### Subject characteristics

**Fig. 1:**
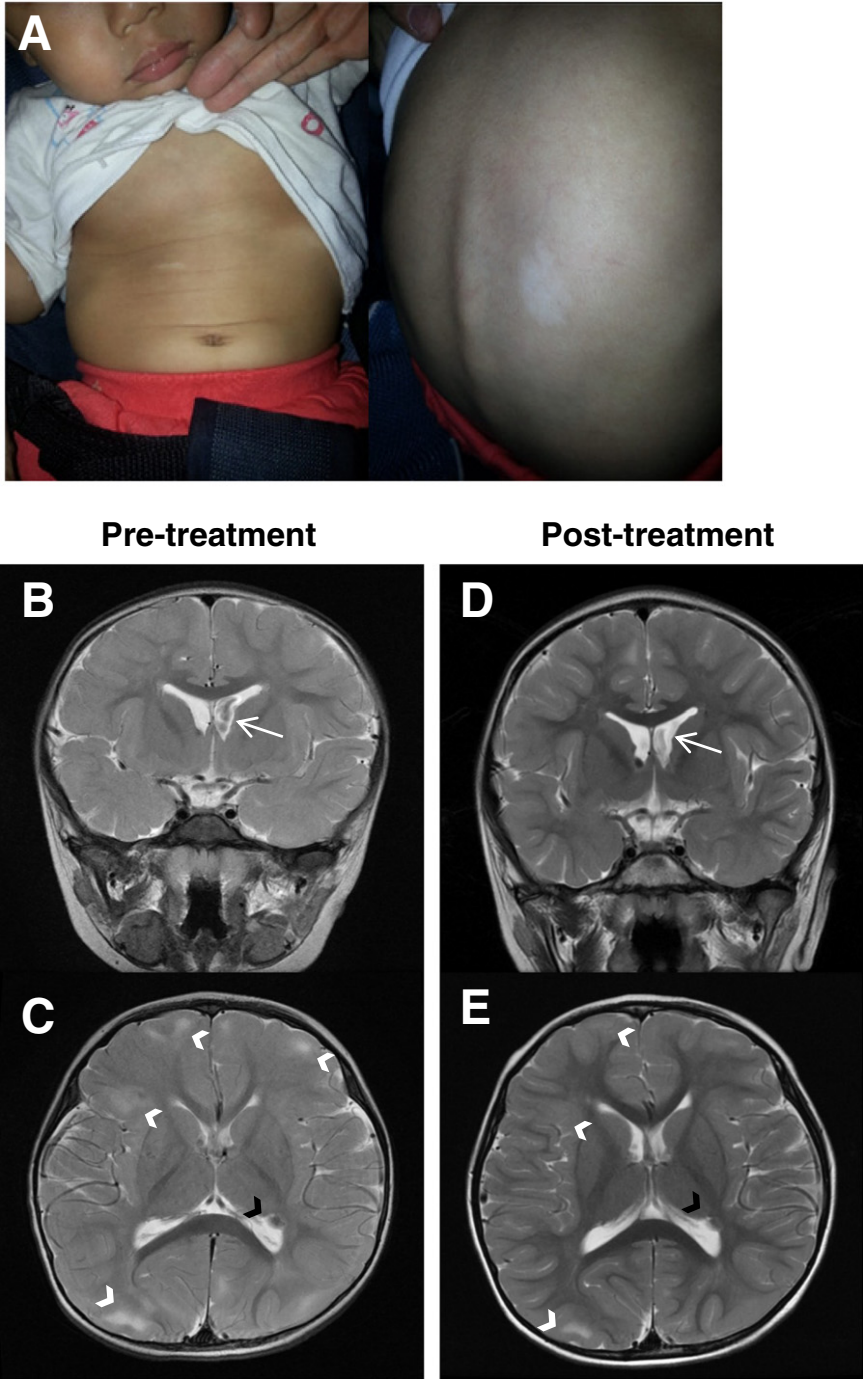
Cutaneous features and brain MRI findings of the patient. **a** Photographs showing several hypopigmented macules on the chest and abdomen (*left*) and on the back (*right*) of the patient. **b-c**. Pre-treatment brain MRI revealing a SEGA (*white arrow*) located at the foramen of Monro within on coronal T2-weighted imaging (**b**), multiple cortical tubers (*white arrowheads*) and a subependymal nodule (*black arrowhead*) within on axial T2-weighted imaging (**c**). **d-e** Post-treatment brain MRI demonstrating size reduction of the SEGA (*white arrow*) (**d**), reduced cortical tubers (*white arrowheads*) and the same size of subependymal nodule (*black arrowhead*) (**e**) compared to pre-treatment MRI

The proband presented intractable epilepsy and severe developmental delay. He was born at 38 weeks gestation by spontaneous vaginal delivery with a birth weight of 2.4 kg after an uncomplicated pregnancy. At the age of 13 months, he experienced his first episode of febrile status epilepticus with a duration of 40 min; at 17 months, a second episode of febrile status epilepticus persisted for more than an hour. Subsequently, he had frequent seizures with or without fever, and was often admitted with status epilepticus. Physical examination identified scattered hypopigmented lesions on the trunk (Fig. 1a). Brain magnetic resonance imaging (MRI) revealed a SEGA (Fig. 1b), cortical tubers and subependymal nodules consistent with TSC (Fig. 1c). The seizures persisted despite the use of vigabatrin and levetiracetam at the maximum tolerated doses.

A baseline psychomotor developmental evaluation was performed when the patient was 45 months of age. The Denver Developmental Screening Test (DDST-II) revealed delayed development in areas of fine motor-adaptive, personal-social, and language skills by 25 months on average. He was not able to get a score in any subtests in the Wechsler Preschool and Primary Scale of Intelligence (WPPSI-III). His social age was 22 months and social quotient was 49.8 by the Social Maturity Scale (SMS). The Childhood Autism Rating Scale (CARS) and Autism Diagnostic Observation Schedule-2 (ADOS-2) showed scores of 35.5 and 20, respectively, which were above the cutoff scores for a diagnosis of autism. His Autism Diagnostic Interview-Revised (ADI-R) scores were also above the autism diagnostic cutoffs in each criterion; 24 in qualitative abnormalities in reciprocal social interaction, 18 for verbal and 12 for non-verbal in qualitative abnormalities in communication, and 5 in repetitive/stereotyped patterns of behavior. The speech and language evaluation revealed that his receptive and expressive language was delayed by 27 months. Other evaluations including ophthalmologic examination, dental examination, ultrasonography and electrocardiography (ECG) of the heart, high resolution computed tomography (HRCT) of the chest, blood pressure, abdominal MRI, and glomerular filtration rate (GFR) testing demonstrated no abnormalities. Evaluations for the diagnosis of TSC and the respective findings are listed in Table 1.

**Table 1:** Evaluations for TSC diagnosis

Organ system	Diagnostic evaluations	Findings
Brain	Brain MRI	Multiple cortical tubers, subependymal nodules and a SEGA
	EEG	Runs of spikes in right fronto-polar region
	DDST-II	Delayed development in areas of fine motor-adaptive, personal-social, and language skills by 25 months on average
	WPPSI-III	Could not get a score in any subtest
	CARS	35.5
	SMS	Social age: 22 months
		Social quotient: 49.8
	Speech and language evaluation	Delayed by 27 months in receptive and expressive language
Eyes	Complete ophthalmologic evaluation including dilated fundoscopy	Unremarkable
Teeth	Detailed dental exam	Unremarkable
Skin	Detailed skin exam	Scattered hypopigmented lesions
Heart	Ultrasonography	Small muscular ventricular septal defect (VSD) in the middle portion of the interventricular septum (IVS), No rhabdomyoma
	ECG	Normal
Lung	HRCT of chest	No lymphangioleiomyomatosis (LAM)
Kidney	Blood pressure	Normal
	Abdominal MRI	No angiomyolipoma or renal cyst
	GFR test	Normal

### Identification of a small deletion variant located in *TSC2* by whole exome sequencing

**Fig. 2:**
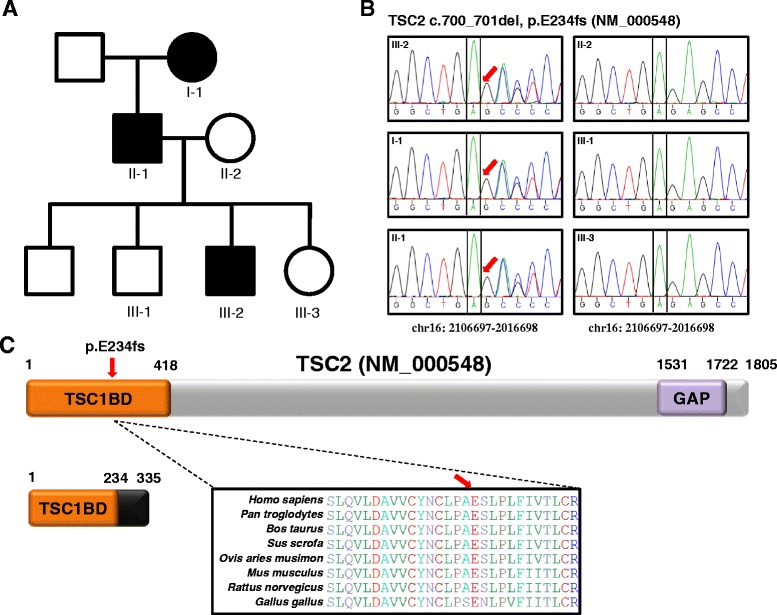
Identification of a small deletion variant in the *TSC2* gene. **a** Pedigree of the affected family. Closed symbols represent affected family members (I-1, II-1, and III-2). Whole exome sequencing was performed for six family members (I-1, II-1, II-2, III-1, III-2, and III-3). **b** Confirmation of the identified small deletion variant in the *TSC2* gene. The two nucleotide deletion (c.700–701del, chr16: 2106697–2016698) identified in the three affected members was validated by Sanger sequencing of the genomic DNA from six family members. The *TSC2* transcript, NM_000548 (RefSeq sequence) with the two nucleotide deletion (c.700–701del; cDNA position 700 and 701) could be translated into a truncated form of the *TSC2* protein because of the frameshift at the amino acid position 234, glutamic acid (p.E234fs). **c** Schematic diagram of the TSC2 protein with the position of the small deletion identified in this study. The variant is located in the TSC1 binding domain (TSC1BD, orange); it is predicted that the variant generates a premature stop codon in the TSC1BD domain. An amino acid multiple sequence alignment of the region near the small deletion for eight different species shows that the region is evolutionally highly conserved. *GAP* GTPase activating protein domain

In the family, three individuals including the grandmother (I-1), father (II-1), and the proband (third son, III-2) met diagnostic criteria for TSC. Definite diagnosis is made by 2 major features or 1 major feature with 2 or more minor features; the grandmother (I-1) and father (II-1) had 3 major features: multiple hypomelanotic macules, angiofibromas, and ungual fibromas. The other family members (including II-2, III-1 and III-3) had no features of TSC (Fig. 2a). In contrast to the proband (III-2), the grandmother (I-1) and father (II-1) had normal intelligence and epilepsy or neuropsychiatric symptoms were not identified.

To confirm a TSC gene mutation, we performed whole exome sequencing for the six family members, generating over 50 million reads (average mean target depth: 65×). In each family member, about 80,000 variants were identified in RefSeq exonic regions. After filtering common variants (see Methods), over 500 variants causing non-synonymous changes, frameshifts, or which were located near splicing sites were identified in each family member. To identify rare and novel causative candidate variants, we selected the variants that were detected in affected (I-1, II-1, and III-2) but not unaffected family members (II-2, III-1, and III-3). A total of 14 variants were identified, five of which were novel (i.e., not reported in dbSNP142) (Additional file 1: Table S1). Among those five, a variant in *GLI2* (NM_004270), c.C2740T (p.R914W) was predicted as deleterious by three different tools (SIFT, PolyPhen-2, and MutationTaster) commonly used to predict the possible impact of amino acid changes. GLI2 is known as a Kruppel-like transcription factor and is associated with cancer progression and metastasis [27]. Notably, one of the novel variants was located in the *TSC2* gene, the mutation of which causes TSC [28] and in which over 700 mutations have been reported [29]. We confirmed that only the affected family members (I-1, II-1, and III-2) carried the variant, identified as a small c.700–701 deletion (chr16: 2106697-2016698) by Sanger sequencing (Fig. 2b). The small deletion variant is located in the TSC1 binding domain and the flanking region of the variant is highly conserved across different species (Fig. 2c). We predicted that the deletion could generate a truncated form of the TSC2 protein because of the introduction of a premature termination codon.

### Everolimus treatment rescued abnormal mTOR pathway activation by the novel *TSC2* frameshift mutation

**Fig. 3:**
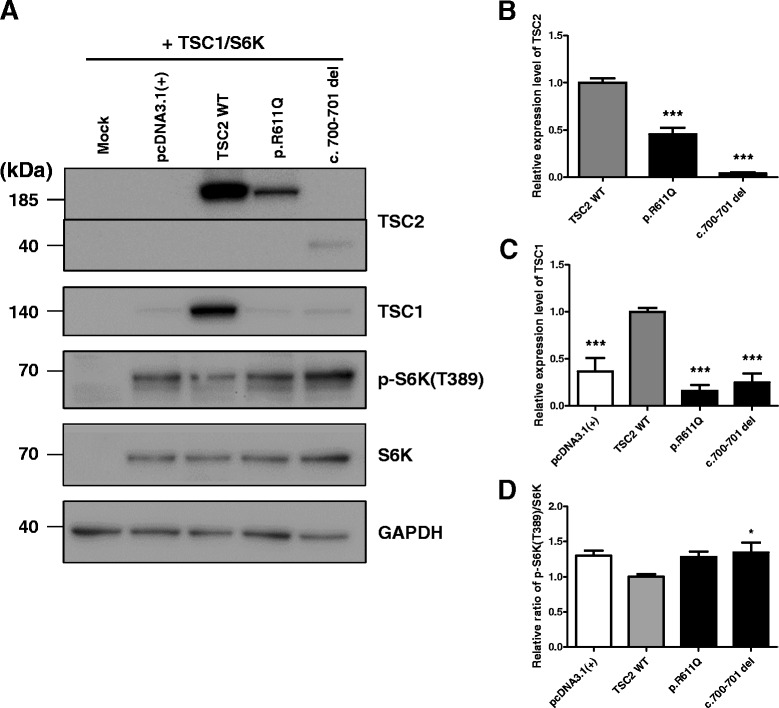
Immunoblot analysis of mTOR pathway activation by TSC2 mutants. **a** Representative immunoblot of S6K1 phosphorylation by novel frameshift TSC2 c.700–701 deletion (del) mutant expression. Note that the c.700–701 del mutant is detected as an approximately 37 kDa-sized band, whereas the wild-type TSC1 is detected as an approximately 200 kDa-sized band. **b**. Relative expression levels of TSC2 mutants in the presence of wild-type TSC1 (One-way ANOVA followed by Dunnett’s test, *** *p* <0.001). **c** Mean TSC1 expression levels in the presence of empty vector and TSC2 mutants relative to wild-type TSC2 (One-way ANOVA followed by Dunnett’s test, *** *p* <0.001). **d** Mean phospho-S6K levels generated by TSC2 variants’ expression relative to wild-type TSC1-TSC2 expression (One-way ANOVA followed by Dunnett’s test, * *p* <0.05). All data are shown as the mean ± SEM of nine independent experiments

To investigate the effect of the novel frameshift mutation of *TSC2* on mTOR pathway activation, we performed a transfection-based immunoblot assay [30, 31]. As predicted, the novel *TSC2* c.700–701 deletion mutant was detected as an approximately 37 kDa-sized band, whereas the wild-type TSC2 was detected as an approximately 200 kDa-sized band on the blot owing to the frameshift resulting in a premature termination of translation (Fig. 3a). The expression level of TSC2 p.R611Q mutant and c.700–701 deletion mutant was reduced compared to TSC2 wild-type (WT: 1.000 ± 0.049, TSC2 p.R611Q mutant: 0.455 ± 0.065, TSC2 c.700–701 deletion mutant: 0.040 ± 0.069, One-way ANOVA followed by Dunnett’s test, all *p* <0.0001, *n* = 9, Fig. 3a and b). Furthermore, the expression level of TSC1 was also reduced compared to that of TSC1 co-transfected with wild-type TSC2 despite the same quantity of *TSC1* DNA construct was transfected (pcDNA3.1(+): 0.367 ± 0.141, WT: 1.000 ± 0.041, TSC2 p.R611Q mutant: 0.161 ± 0.061, TSC2 c.700–701 deletion mutant: 0.247 ± 0.100, One-way ANOVA followed by Dunnett’s test, all *p* < 0.0001, *n* = 9, Fig. 3a and c), which is consistent with previous reports [30, 31]. In contrast, the phospho-S6K (T389) level was significantly increased in the novel mutant group compared to control wild-type group (WT: 1.000 ± 0.034, TSC2 c.700–701 deletion mutant: 1.343 ± 0.145, One-way ANOVA followed by Dunnett’s test, *p* <0.05, *n* = 9, Fig. 3a and d), suggesting that the expression of the TSC2 c.700–701 deletion mutant results in hyperactivation of the mTOR signaling pathway [30].

**Fig. 4:**
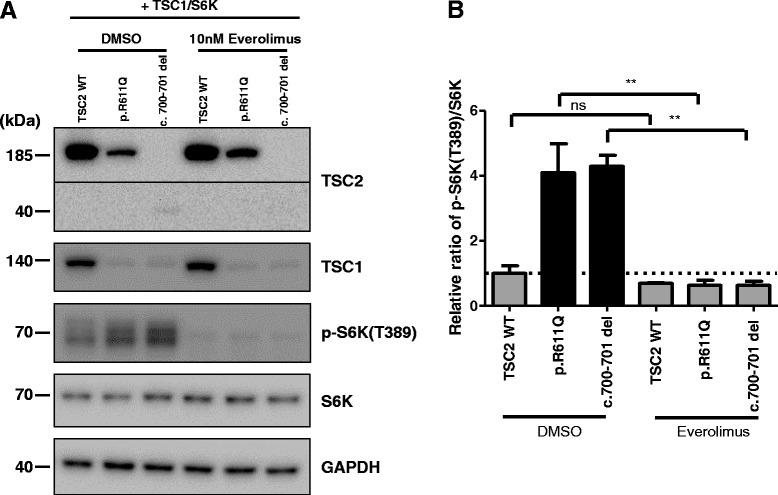
Rescue of elevated mTOR pathway activation by everolimus treatment. **a** Representative immunoblot of reduced S6K1 phosphorylation by everolimus treatment. **b** Mean phospho-S6K levels generated by TSC2 variants’ expression relative to wild-type TSC1-TSC2 expression in the presence of vehicle (DMSO) or everolimus (One-way ANOVA followed by Bonferroni’s test, ** *p* <0.01). All data are shown as the mean ± SEM of three independent experiments

Next, to investigate whether an mTOR inhibitor could rescue elevated mTOR pathway activation by the novel TSC2 frameshift mutation, we treated 10 nM everolimus to the *TSC2*-transfected HEK293T cell cultures and performed immunoblot assays. Everolimus significantly reduced the elevated phospho-S6K (T389) level in p.R611Q mutant group (4.096 ± 0.884 to 0.637 ± 0.156, One-way ANOVA followed by Bonferroni’s test, *p* <0.01, *n* = 3) and novel TSC2 mutant group (4.285 ± 0.343 to 0.639 ± 0.114, One-way ANOVA followed by Bonferroni’s test, *p* <0.01 *n* = 3, Fig. 4a & b). The phospho-S6K (T389) level in the everolimus-treated wild-type group was not significantly reduced (1.000 ± 0.241 to 0.693 ± 0.022, One-way ANOVA followed by Bonferroni’s test, *n* = 2, Fig. 4a and b).

### Everolimus dosing and tolerability

Confirming that the mutation caused hyperactivation of mTOR signaling encouraged us to treat the patient with the mTOR inhibitor everolimus. Everolimus was thus administered when the patient was 45 months of age. The body surface area was 0.86 m^2^ and the median maintenance dose of everolimus was 5.81 mg/m^2^/day. Serum levels during the maintenance dose were between 5 and 15 ng/mL. During the study, the patient exhibited aggression and irritability for several weeks. No severe or life-threatening side effects related to treatment occurred. The patient recovered without dose adjustment and was able to continue everolimus treatment.

### Everolimus treatment reduced size of SEGA

There was a size reduction of SEGA on post-treatment brain MRI after 4 months compared to baseline. Pre-treatment coronal T2-weighted imaging shows a 1.5 cm sized hypointense SEGA located at the foramen of Monro (Fig. 1b). Post-treatment coronal T2-weighted imaging reveals a reduced size of SEGA with a maximum diameter of 1.1 cm (Fig. 1d).

Multiple cortical tubers and a subependymal nodule within on pre-treatment MRI (Fig. 1c) demonstrated reduced cortical tubers and the same size of subependymal nodule in post-treatment MRI (Fig. 1e).

### Everolimus treatment reduced seizure frequency and duration

**Fig. 5:**
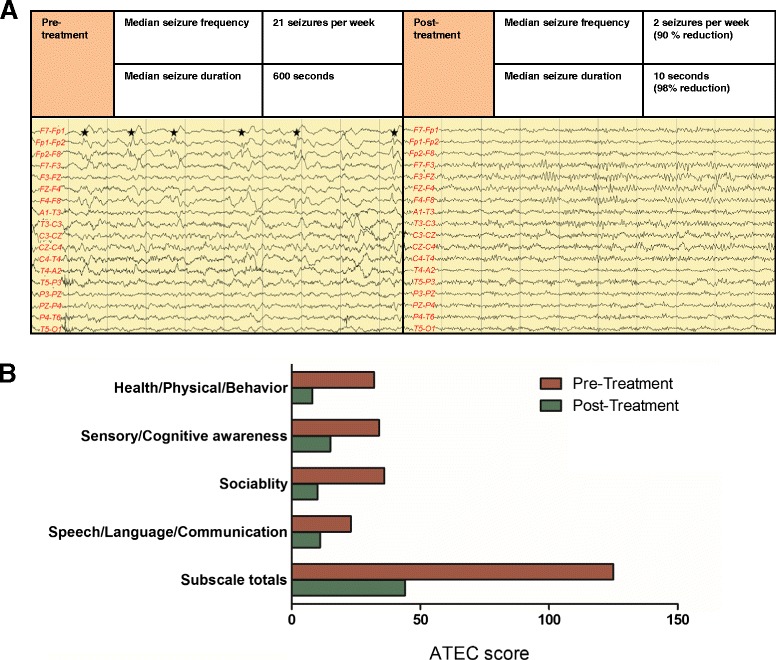
Effect of everolimus treatment on seizure and general behavioral features. **a** The median seizure frequency and duration decreased compared to that prior to everolimus treatment. EEG showed run of spikes in the right fronto-polar region prior to everolimus treatment (*left side*), whereas no epileptiform discharge was observed following treatment (*right side*). **b** In the evaluation of the improvement in general and autistic behavioral features, ATEC showed a decrease in the scores in all four categories (Speech/Language/Communication, Sociability, Sensory/Cognitive awareness, and Health/Physical/Behavior) after everolimus treatment compared to pre-treatment

After treatment with everolimus, the seizure frequency and duration in the patient was significantly reduced. Vigabatrin and levetiracetam were withdrawn by the parents 1 month after treatment and the patient was seizure free for more than 3 months. Although seizures occasionally recurred during treatment with everolimus for which vigabatrin was administered, the median seizure frequency was decreased by 90 % (pre- vs. post-treatment, 21 vs. 2 seizures per week) and the median seizure duration was also decreased by 98 % (600 vs. 10 s) compared to that prior to everolimus treatment (Fig. 5a). In addition, electroencephalography (EEG) showed run of spikes in the right fronto-polar region prior to everolimus treatment, whereas no epileptiform discharge was observed following treatment (Fig. 5a).

### Everolimus treatment improved general behavioral deficits and autistic phenotypes

Everolimus treatment had a great impact on reducing behavioral deficits and improved cognition, attention, social interaction, and language development in the patient. Just 1 day after the initiation of treatment, he started to understand and follow some verbal commands. After several days, episodes of urinary incontinence no longer occurred and the patient urinated in the toilet for the first time. Repetitive motions such as swaying or twirling and obsession with running water disappeared. At 1 month after treatment, the patient began to use two words sentences.

The parents were also asked to provide the Autism Treatment Evaluation Checklist (ATEC) scores prior to and after 1 year of the intervention. The ATEC has been successfully used to measure treatment effects and progress over time in several studies on ASD [32–34]. Following the initiation of treatment, the ATEC score was markedly decreased indicating a decrease in severity of ASD symptoms (Fig. 5b). Subscale totals more than 104 are considered to indicate very severe autism as they are in the 90th percentile; patients with scores less than 50 are considered to have good chances of being semi-independent and are within the 30th percentile level. A lowered score by over 50 points is considered to represent marked improvement following autism treatment. Here, the subscale totals of the patient decreased from 127 prior to treatment to 44 following treatment initiation; substantial decreases were observed following treatment initiation in all four of the ATEC categories. The Speech/Language/Communication ATEC scores decreased from 23 prior to treatment to 11 following treatment initiation; Sociability ATEC scores decreased from 36 to 10; Sensory/Cognitive Awareness ATEC scores decreased from 34 to 15; and the Health/Physical/Behavior ATEC scores decreased from 32 to 8. The efficacy of everolimus on general and autistic behavioral deficit were not diminished over up to 2 days without medication, but it remains to be systematically determined how long the effect of everolimus would last following treatment cessation.

## Discussion

In the present study, we describe a case of a Korean family in which three of the members have been diagnosed with TSC. Among the affected members, only a boy (III-2 in Fig. 2a) showed diverse behavioral and cognitive deficits including autistic phenotypes. Whole exome analysis revealed a novel deletion mutant in the *TSC2* gene (*TSC2* c.700–701 del).

We found that the expression of the newly identified mutant TSC2 protein enhanced the activation of the mTOR signaling pathway in HEK293T cells, suggesting that the novel mutation represents a loss-of-function mutation as are other *TSC* mutations associated with TSC [28]. In our biochemical analyses, we found that the protein expression level of the deletion mutant was significantly decreased (Fig. 3a, b). The reduced TSC2 c.700–701 del mutant expression level might be caused by nonsense-mediated mRNA decay as a quality-control mechanism [35–37]. In addition, it has been reported that the TSC1-TSC2 interaction is important for stability of both TSC1 and TSC2 [38, 39]. In this patient, it is hypothesized that the TSC1 protein would be destabilized since the mutation in TSC2 is located within the essential region for interacting with TSC1 (amino acids 1–900) [31, 40] (Fig. 3a, c). Furthermore, the loss of the C-terminal GTPase activating protein (GAP) domain of TSC2 would also be predicted to disrupt the action of the TSC1-TSC2 complex on the GTPase Ras homolog expressed in brain (RHEB), thus activating RHEB–GTP-dependent stimulation of the mammalian target of rapamycin complex 1 (mTORC1) [41]. Therefore, one of the downstream mTORC1 targets, S6K T389 phosphorylation, would be elevated (Fig. 3a, d). Additionally, we examined the effect of everolimus treatment on elevated S6K T389 phosphorylation induced by *TSC2* c.700–701 del mutation and found that everolimus could reduce the activation of the mTOR signaling pathway (Fig. 4a, b).

The efficacy of mTOR inhibitors for treating TSC-associated phenotypes has been demonstrated in multiple animal models. For example, Ehninger and colleagues showed that rapamycin treatment reversed the deficits in learning and in hippocampal synaptic plasticity in *Tsc2*^+/−^ mice [18]. Rapamycin or everolimus (RAD001, 40-O-(2-hydroxyethyl)-rapamycin) treatment rescued lethality, brain enlargement, and hyperactivity in *Tsc1* conditional knockout mice [18, 19]. Notably, deleting *Tsc1* in mouse cerebellar Purkinje cells resulted in autistic-like behaviors, which can also be reversed by rapamycin treatment [20]. In addition, deficits in social interaction in *Tsc1*^+/−^ and *Tsc2*^+/−^ mice were also reversed by rapamycin treatment [26]. These animal studies support the hypothesis that mTOR activation is responsible for the ASD-associated phenotypes in TSC [16, 26, 42] and thereby mTOR inhibition might be an effective treatment strategy for ASD symptoms in TSC patients [10, 25].

In our study, everolimus treatment resulted in a rapid and marked reduction of behavioral deficits and improved cognition, attention, social interaction, and language development in the patient. As indicated by changes in the ATEC subscale scores, the classification of severe autism with scores in the 90th percentile was modified to that of semi-independent autism with scores in the 30th percentile level. The total score dropped by 83 points, which is considered to be a remarkable improvement for an autism treatment. In addition to the improvement of autistic features, everolimus showed marked effectiveness in mediating the intractable seizures of TSC, such that the seizure frequency and the median seizure duration decreased by over 90 % in the patient. However, we cannot insist that everolimus would have therapeutic effect specifically on ASD or epilepsy in patients with TSC induced by *TSC2* c.700–701 del mutation because grandmother and father carrying *TSC2* c.700–701 del mutation has normal intelligence without epilepsy or neuropsychiatric symptoms and we could not suggest an evidence of genotype (*TSC2* c.700–701 del mutation)-phenotype correlation in TSC patient. Therefore, we consider that individuals affected by *TSC2* c.700–701 del mutation could exhibit a high variability in clinical findings and further studies are needed to identify a key mechanism underlying the therapeutic effect of everolimus on TSC symptoms with ASD.

Nevertheless, early recognition of ASD in patients with TSC and proper management with everolimus might give a life-enhancing effect on the long-term outcome of the disorder. Consistent with our findings, Ishii and colleagues recently reported that everolimus treatment improved the irritability, stereotypic behavior, inappropriate speech, and social behavior in a 27 year-old female patient with TSC [43]. In addition, Wheless showed that everolimus reduced the seizure frequency in a 13-year-old girl with TSC-associated epilepsy after 1.5 years of treatment [44]. Kruger and colleagues also demonstrated the antiepileptic effect of everolimus in the majority of TSC patients [45]. Focal cortical malformations such as cortical tuber are highly associated with epileptogenesis and epilepsy related to cortical tuber is often refractory to antiepileptics [46]. mTOR inhibitor treatment also reduces the size of cortical tubers in TSC patients [47].

Together with our results, these studies strongly support that the inhibition of mTOR signaling represents a valid treatment strategy for the neurological and psychiatric manifestations associated with TSC. As additional clinical trials to test the efficacy of everolimus for treating the psychiatric symptoms associated with TSC have been completed or launched [10, 17], it will likely soon be determined whether the use of everolimus or other mTOR inhibitors will be approved to treat the features of ASD associated with TSC in the clinic.

In summary, we identified a novel small *deletion* mutation in *TSC2* associated with severe TSC in a Korean family that enhances the activation of mTOR signaling in vitro. Moreover, everolimus treatment showed not only reduction in SEGA size, but improved behavioral deficits including autistic phenotypes and seizures in the patient.

## Methods

### Participants

The study participants consisted of a three-generation family whose members were diagnosed with TSC. TSC showed autosomal dominant inheritance throughout the family history; the grandmother was the first affected family member. Six individuals including the grandmother, father, mother, second and third sons, and daughter participated in this study.

### Diagnostic evaluation

#### Diagnosis of TSC

Brain MRI, EEG, complete ophthalmologic evaluation including dilated fundoscopy, detailed dental examination, careful skin examination with a Wood’s lamp, ultrasonography and ECG of the heart, HRCT of the chest, blood pressure, abdominal MRI, and GFR tests were performed. In addition to genetic diagnosis (described below), clinical diagnosis was made according to the “Tuberous Sclerosis Complex Diagnostic Criteria Update: Recommendations of the 2012 International Tuberous Sclerosis Complex Consensus Conference” [48].

#### Measures and procedure

A baseline psychomotor developmental evaluation including the DDST-II, WPPSI-III, CARS, SMS, and speech and language evaluations was performed prior to everolimus treatment. The ADOS and ADI-R were conducted by an experienced examiner. For ADOS, module 1 for 31 months and older children with pre-verbal/single words was applied. The ATEC was also implemented for the severity evaluation of autistic features prior to and following completion of the intervention.

### Intervention

Everolimus treatment was initiated, at 5 mg/m^2^/day, administered once daily in the morning and rounded to the nearest 2.5 mg/dose. A serum everolimus level was obtained at every 2 weeks, and the treatment dose was adjusted to obtain a target range between 5 and 15 ng/ml. No further adjustments were made after the stable target range was obtained, unless a severe side effect or considerable weight gain was noticed. Safety and efficacy of treatment was observed for 1 year.

### Whole exome sequencing and variant calling

Whole blood was obtained from the family members (the grandmother, father, mother, elder brother, and little sister of the patient) after informed consent for the protocol (KNUH 2013-07-011-004) guided by the KNUH IRB was obtained. Genomic DNA was extracted from the blood from each subject and about 3 μg genomic DNA from each family member was subjected to library preparation and exome capture following the Agilent SureSelect Human All Exon v4 Illumina Paired-End Sequencing Library Prep Protocol (Agilent Technologies, Santa Clara, CA, USA). The prepared sequencing library was used to perform sequencing on an Illumina HiSeq-2000 system (San Diego, CA, USA) as a 100 bp paired-end run. In each sample, about 51–65 million reads were generated. The raw sequencing reads were checked and trimmed using the sickle program (version 1.33) to ensure the quality of the raw reads. Processed reads were mapped to the reference human genome sequence GRCh37 using the Burrow-Wheeler Aligner (BWA, version 0.7.10) [49]. To reduce the potential bias problems caused by the sequencing processes, the mapped duplicated reads were marked using Picard (version 1.118). Insertion and realignment (INDEL) realignment and base quality recalibration were performed using GATK (version 3.2.2) [50]. The average of the mean target coverage was 65 and 85 % of the target exome was covered to 20×. Using the alignments, both small nucleotide variants (SNVs) and small INDELs were called by the GATK HaplotypeCaller and called variants were filtered using the GATK variant quality score recalibration process. Finally, for each family member, 84,356–86,901 variants were obtained. The variants were further filtered to exclude those included in dbSNP142, 1000 Genomes Project (Oct 2014), NHLBI-ESP project with 6500 exomes, or ExAC 65,000 exomes at the level of 5 % minor allele frequency (MAF) by ANNOVAR [51]. In addition, variants were filtered out if they existed in in-house genome and exome databases.

### Sanger sequencing

To validate the small deletion variant in the *TSC2* gene, Sanger sequencing was performed. The target site of the variant and the flanking sequences of the DNA template from each family member were amplified with specific primers (forward primer 5′-ACAGTGACAGGGACGTCAGGTG-3′ and reverse primer 5′-ACAACCATTCATGGGAGACAGGA-3′) and the amplified products were directly sequenced on an ABI PRISM 3730 automated sequencer (Applied Biosystems, Foster City, CA, USA). The results were compared with the reference human genome sequence, GRCh37, to confirm the deletion variant.

### DNA constructs

Full-length *TSC1* with a Myc-tag at the C-terminus, wild-type *TSC2*, and the pathogenic *TSC2* mutation p.R611Q were kindly provided by Dr. Mark Nellist (Eramus Medical Centre, The Netherlands). pcDNA-Myc S6K1 was a gift from Jie Chen (Addgene plasmid #26610, Cambridge, MA, USA) [52]. The novel *TSC2* c.700–701 deletion construct was derived from the wild-type *TSC2* construct by site-directed mutagenesis using a QuikChange II XL Site-Directed Mutagenesis Kit (Agilent Technologies, Santa Clara, CA). N-terminal HA-tagged wild-type *TSC2*, *TSC2* p.R611Q, and *TSC2* c.700–701 deletion mutants were subcloned into the pcDNA3.1(+) vector using *Bam*HI and *Xho*I sites. The complete open reading frame of the each construct was verified by sequencing. Construct DNA were prepared using the PureYield™ Plasmid Midiprep System (Promega, Madison, WI, USA).

### Antibodies

Phospho-p70 S6 kinase Thr389 rabbit monoclonal (108D2 for Fig. 3 or 9205S for Fig. 4), Myc-tag mouse monoclonal (9B11), and HRP-conjugated anti-rabbit IgG (#7074) antibodies were purchased from Cell Signaling Technology (Danvers, MA, USA). HRP-conjugated goat anti-rat IgG (#AP183P), HRP-conjugated anti-mouse IgG (H+L) (#SA001-500), and anti-HA-Fluorescein, high affinity (#11 988 506 001) antibodies were obtained from Millipore (Darmstadt, Germany), GenDEPOT (Barker, TX, USA), and Roche (Basel, Switzerland), respectively.

### Immunoblotting

A transfection-based immunoblot assay for the functional assessment of the TSC2 variants was performed as described previously [30, 31]. HEK293T cells were plated on a 12-well plate and grown overnight in Dulbecco’s modified eagle’s medium (DMEM) (Hyclone, Logan, UT, USA) with 10 % fetal bovine serum (FBS). Confluent cells (70–90 %) were transfected with 0.4 μg *TSC2*, 0.8 μg *TSC1*, and 0.2–0.3 μg *S6K1* constructs using 4 μL Lipofectamine® 2000 (Thermo Fisher Scientific, Waltham, MA, USA) in Opti-MEM. The transfected medium was replaced with DMEM with 10 % FBS at 4 h after transfection. And then, everolimus (10 nM, LC Laboratories, Woburn, MA, USA) was added and incubated for another 20 h. The transfected cells were harvested, washed with cold phosphate-buffered saline, and lysed with 200 μL RIPA buffer (150 mM NaCl, 1.0 % Triton X-100, 0.5 % sodium deoxycholate, 0.1 % SDS, and 50 mM Tris-Cl, pH 8.0) containing a protease inhibitor cocktail and a phosphatase inhibitor cocktail (Roche). After full-speed centrifugation for 15 min at 4 °C, the supernatant fractions were quantified using a Thermo Scientific™ Pierce™ BCA Protein Assay kit. The protein samples (4.5 μg each) were electrophoresed on Bolt® 4–12 % Bis-Tris Plus Gels (Thermo Fisher Scientific) and transferred to PVDF membranes (Millipore) according to the manufacturer’s recommendations.

The blots were blocked for 10 min at room temperature with either 5 % Blotto, non-fat dry milk (sc-2325, Santa Cruz Biotechnology, Dallas, TX, USA) or 5 % bovine serum albumin in TBST (Tris-buffered saline plus 0.1 % Tween-20). Blots were incubated overnight at 4 °C with the following primary antibodies: 1:1000 dilutions of rabbit monoclonal anti-p-S6K (T389) or rat anti-HA antibody, or 1:10,000 dilutions of mouse monoclonal anti-myc tag antibody. After washing three times for 10 min in TBST, the blots were incubated for 2 h at room temperature with 1:5000 dilutions of secondary antibodies. After washing three times for 10 min in TBST, the blots were scanned using a ChemiDoc™ XRS System (Bio-Rad, Hercules, CA, USA) after applying detection reagents. To estimate the ratio of p-S6K (T389) to total S6K, the scans were analyzed using Image Lab™ Software (Bio-Rad).

### Statistics

One-way analysis of variance (ANOVA) with appropriate post-hoc tests (Dunnett’s or Bonferroni’s test) was used for western blotting results. Each “n” indicates an independent set of experiment. Differences are considered significant at the level of *p* <0.05.

## Additional file

Additional file 1: Table S1. A list of identified variants that were not detected in unaffected members but detected in affected members (PDF 35 kb)

## Abbreviations

ASD: autism spectrum disorder
TSC: tuberous sclerosis
mTOR: mammalian target of rapamycin
SEGA: subependymal giant cell astrocytomas
MRI: magnetic resonance image
DDST-II: Denver Developmental Screening Test
WPPSI-III: Wechsler Preschool and Primary Scale of Intelligence
SMS: Social Maturity Scale
CARS: Childhood Autism Rating Scale
ADOS-2: Autism Diagnostic Observation Schedule-2
ADI-R: Autism Diagnostic Interview-Revised
ECG: electrocardiography
HRCT: high resolution computed tomography
GFR: glomerular filtration rate
EEG: electroencephalography
ATEC: Autism Treatment Evaluation Checklist
GAP: GTPase activating protein
RHEB: Ras homolog expressed in brain
mTORC1: mammalian target of rapamycin complex 1
INDELs: insertions and deletions
SNVs: small nucleotide variants
MAF: minor allele frequency
DMEM: Dulbecco’s modified eagle’s medium
