# Supplementary material for: Everolimus improves neuropsychiatric symptoms in a patient with tuberous sclerosis carrying a novel TSC2 mutation
Source: Mol Brain. 2016 May 23;9:56. doi: 10.1186/s13041-016-0222-6 (PMC4878062; doi:10.1186/s13041-016-0222-6)
Supplement: Additional file 1: Table S1. — A list of identified variants that were not detected in unaffected members but detected in affected members (PDF 35 kb) [file 13041_2016_222_MOESM1_ESM.pdf]

Table S1. A list of identified variants that were not detected in unaffected members but detected in affected members

Chromosome	Position	Reference	Alternative	dbSNP142	Gene	Functaional category	SIFT_pred[@]	Polyphen2_HDIV _pred[&]	MutationTaster_pred[#]
11	95825277	G	C	.	*MAML2*	ns SNV[a]	T	B	D
16	11773400	C	T	rs184422078	*TXNDC11*	ns SNV	T	B	N
16	14042187	G	A	rs150077735	*ERCC4*	ns SNV	T	P	D
16	2051127	T	G	rs541079516	*ZNF598*	ns SNV	T	D	D
16	2106697	GA	-	.	*TSC2*	frameshift deletion	.	.	.
16	5057470	C	T	rs200260160	*SEC14L5*	ns SNV	D	B	D
16	56700837	C	T	rs202203178	*MT1G*	ns SNV	T	D	D
16	66918288	G	T	.	*PDP2*	ns SNV	T	P	N
16	70154622	G	C	.	*PDPR*	ns SNV	T	B	D
2	120397446	G	T	rs147638703	*CFAP221*	ns SNV	T	B	N
2	121746230	C	T	.	*GLI2*	ns SNV	D	D	D
2	128471559	G	A	rs185615277	*WDR33*	ns SNV	T	B	D
3	157081492	C	T	rs143505735	*VEPH1*	ns SNV	T	P	D
4	5570315	G	A	rs182298453	*EVC2*	ns SNV	T	B	N

@: D. Deleterious; T, tolerated

&: D, probably damaging; P, possibly damaging; B, benign

#: D, disease causing; N, polymorphism

a: ns SNV, nonsynonymous SNV
